# Persistence and Adherence during the First Six Months of Tocilizumab Treatment Among Rheumatoid Arthritis Patients in Routine Clinical Practice in Greece. Results from the Single Arm REMISSION II Study (NCT01649817)

**DOI:** 10.31138/mjr.30.3.177

**Published:** 2019-09-30

**Authors:** Theodora E. Markatseli, Athina Theodoridou, Marina Zakalka, Eftychia Koukli, Eva Triantafyllidou, Sotiris Tsalavos, Alexandros Andrianakos, Alexandros A. Drosos

**Affiliations:** 1 Rheumatology Clinic, Department of Internal Medicine, Medical School, University of Ioannina, Ioannina, Greece,; 2 Academic Clinical Fellow, Rheumatology Unit, D’ Internal Medicine Department, Hippokrateion University Hospital, Thessaloniki, Greece,; 3 Research Fellow, Division of Clinical Immunology, 1^st^ Internal Medicine Department, AHEPA University Hospital, Thessaloniki Greece,; 4 Private Practice, Greece,; 5 Roche Hellas, Marousi, Greece,; 6 Iaso General Hospital, Athens, Greece

**Keywords:** rheumatoid arthritis, tocilizumab, clinical practice, private practice, Greece

## Abstract

**Objective/Aim:**

One of the most important factors that affect a treatment’s performance in rheumatoid arthritis (RA) is adherence to medications. According to literature, there are several reasons for non-adherence in RA patients with some of them being related to a specific patient profile of the study population. In this study, we investigated persistence to intravenous tocilizumab (TCZ) therapy in RA during routine clinical practice in Greece and identified causes for non-adherence.

**Methods:**

183 RA patients who mostly attended private practice Rheumatologists and received intravenous TCZ treatment at a schedule of 1 infusion per 4-weeks in the first 6 months were recorded retrospectively.

**Results:**

Persistence estimated rate to TCZ therapy was 92.0% for patients that received 6 infusions and 83.4% for patients that received 7 infusions of TCZ. Potential factors that influence persistence to therapy were the occurrence of adverse events and response to the therapy. The main reasons for non-adherence to TCZ therapy were non-medically related with the most common being drug supply issues. The 6-month mean change from baseline in DAS28-ESR after initiation of TCZ therapy was −1.3, and the mean CDAI dropped from 29.6 at baseline to 16.7 at 6 months. Good/Moderate response was achieved by 89.1% of patients and remission by 23.5%. The safety profile was similar to that observed in other TCZ trials with the most common being infections, hematologic manifestations and musculoskeletal disorders.

**Conclusion:**

Overall, persistence to therapy appeared to be high in the rheumatology private practice setting and non-adherence to the TCZ treatment schedule is attributed mainly to non-medical reasons.

## INTRODUCTION

Rheumatoid arthritis (RA) is a chronic systemic inflammatory auto-immune disease responsible for articular and extra-articular signs and symptoms. Due to articular involvement, over one third of RA patients eventually experience work disability.^1^ In the past, treatment was only symptomatic, intended to reduce pain and inflammation and was based on the use of non-steroidal anti-inflammatory drugs (NSAIDs) or corticosteroids. With the introduction of conventional synthetic disease-modifying anti-rheumatic drugs (csDMARDs) and biologic DMARDS (bDMARDs) treatment management of the disease has evolved over the years focusing on achieving of clinical remission.^2^

Interleukin 6 (IL-6) is a pleiotropic pro-inflammatory cytokine, described as inflammation mediator.^3^ In RA patients, levels of IL-6 are increased in the synovial fluid and tissue and they are correlated with C-reactive protein (CRP) and disease activity.^4^ Tocilizumab (TCZ) is a recombinant humanized anti-IL-6 receptor monoclonal antibody approved in RA and in juve nile idiopathic arthritis.^5^ It has a unique combination of rapid onset of action and effectiveness which is increasing during time in several types of patient populations such as patients with intolerance to methotrexate (MTX), inadequate response to csDMARDs and inadequate response to anti-tumour necrosis factors (TNFs).^6–9^ Despite our knowledge of the clinical benefits of TCZ from phase II–IV controlled studies, such data do not reflect daily clinical practice. Furthermore, data on the use of TCZ in Southern Europe across various clinical settings is limited. This is important in view of the variations in disease severity across different ethnic backgrounds and clinical settings.^10,11^ Real World Data (RWD) which reflect the use in routine clinical practice have been emerging, thus providing valuable insights on the efficacy and safety of TCZ as well as on the ethnic characteristics of patients receiving TCZ therapy.^12^ Along with published data from country registries with similar ethnic characteristics and backgrounds, our understanding on the use of TCZ and its clinical effectiveness is becoming more concrete.^13,14^ In the REMISSION II study, we sought to investigate retrospectively the persistence and adherence to TCZ therapy in the first 6 months of the daily clinical routine, concentrating on private practice rheumatologists in Greece.

## PATIENTS AND METHODS

The REMISSION II (ML 28258) study is a retrospective, open-label, multicentre, non-interventional study. A total of 183 patients were enrolled from 2 coordinating rheumatology clinics and 24 private rheumatology practitioners in Greece, according to ICH GCP guidelines, the Declaration of Helsinki and the approval of the protocol by the local Ethics Committees. Signed informed consent was obtained by all patients prior to enrolment in the study. Case report forms (CRFs) were recorded from May to October 2012.

Adult patients with diagnosis of RA by the American College of Rheumatology (ACR) 1987 criteria that received a minimum of one dose of TCZ (RoActemra®), at least 6 months prior to enrolment, and, according to the SPC and the local clinical practice, were eligible to participate in the study. Patients with rheumatic autoimmune disease other than RA, that refused or were incapable to give written consent or participating in an interventional study, were excluded.

Patients received TCZ at a dose of 8mg/kg every 4 weeks intravenously (IV). The primary endpoint was persistence to the 6-month treatment with TCZ. Patients were considered to be persistent if they had received at least 6 infusions within 6 months of therapy. Secondary endpoints were the percentage of patients who discontinued treatment and clinical effectiveness of TCZ therapy. Adherence to TCZ therapy was defined as the administration of an infusion at exactly 4 weeks from the previous one.

In addition to the objectives, the following data were collected as per local clinical practice: patient demographics, body-mass index (BMI), duration of RA prior to TCZ initiation, comorbidities, laboratory test results, reasons for treatment discontinuation and reasons for non-adherence to the scheduled infusion. Disease clinical assessment before therapy initiation and at 6 months was carried out by the investigators. Response to TCZ treatment was evaluated using the European League Against Rheumatism (EULAR) criteria at 6 months. RA disease activity was assessed by the 28-joint disease activity score taking into account the Erythrocyte Sedimentation Rate (DAS28-ESR) and the Clinical Disease Activity Index (CDAI).

The study was sponsored by Roche (Hellas) S.A., and registered at ClinicalTrials.gov , NCT01649817.

## STATISTICAL ANALYSIS

In accordance with the primary objective, the estimation of sample size was based on the assumption that 80% of patients (min-max limits of the analysis 70 – 90%) of the overall population had to remain under treatment continuously for at least 6 months. The sample required was set at 228 patients using the formula N= 4Z_a_^2^P(1−P)/W^2^ (Confidence level) = 95%. Proportions of patients in the groups were compared by a chi-squared method. The statistical significance of the mean differences in efficacy endpoints between visits has been examined with the use of the paired t-test. A value of p<0.05 was chosen as indicative of statistical significance.

Kaplan–Meier methodology was used to estimate persistence to therapy for all patients, as well as for subgroups of patients stratified by type of TCZ therapy, AE occurrence, response to therapy and remission status. The log rank test was used for group comparison and a p<0.05 denoted statistical significance. Exploratory Cox analysis was undertaken to investigate factors that were prognostic for therapy discontinuation. Investigated covariates included age (18 – 60 years vs over 60 years), gender (female vs male), TCZ therapy (monotherapy vs combination therapy [TCZ + csDMARD]), BMI (less than 25 kg/m^2^ vs equal and over 25 kg/m^2^), RF/anti-CCP status (both negative vs at least one positive) and prior bDMARD therapy (0 to 1 vs 2 or more). The level of statistical significance was set at 0.05 (two-sided test) Statistical analyses were performed with SAS, v9.4 (SAS Institute Inc.)

## RESULTS

A total of 183 patients that received TCZ from 26 sites in Greece were enrolled in the study. Patient characteristics, including comorbidities, baseline clinical evaluations and laboratory parameters as well as previous lines of therapy are shown in *Table 1*. The median age of patients was 59 years, 43% being over 60 years, with a very high prevalence of females to males. Mean duration of RA prior to TCZ therapy was 8.7 years. Interestingly, the majority of the patient population enrolled in this study was overweight with an average BMI of 28.1 kg/m^2^. This finding seems to be country specific as 68% of the RA patients who have been treated with biologics are overweight and obese.^15^

**Table 1.** Baseline demographics and disease characteristics of patients with rheumatoid arthritis participating in the study.

**Characteristics**	**Patients receiving TCZ therapy n = 183**
**Age**, yrs, mean (SD)	59 (10.7)
Median, yrs (range)	59 (31–88)
>60 yrs, n (%)	79 (43.2)
**Gender**, female/male (%)	161/22 (88/12)
**Weight**, kgr, mean (SD)	75.1 (15.7)
**BMI**, kgr/m^2^, mean (SD)	28.1 (5.2)
**Duration of RA from TCZ initiation**, yrs, mean	8.7
***Comorbidities, n (%)***	
Hyperlipidaemia	99 (54.1)
Elevated triglycerides	50 (27.3)
Cardiovascular disorders	68 (37.2)
Thyroid disorders	34 (18.6)
Diabetes	30 (16.4)
Hepatic disorders	10 (5.5)
GI perforation or ulceration or diverticulitis	8 (4.4)
***Clinical parameters***	
TJC, *(n = 166)*, mean (SD)	10.9 (5.8)
SJC, *(n = 166)*, mean (SD)	8.0 (5.9)
*RF, ACPA, n (%)*	
Negative	49 (26.8)
At least one positive	130 (71.0)
Unknown	4 (2.2)
DAS28-ESR, *(n = 152)*, mean (SD)	5.8 (0.9)
DAS28-CRP, *(n = 122)*, mean (SD)	4.8 (0.9)
***Laboratory parameters*, mean value (SD)**	
ESR, (n = 181), mm/1h	47.4 (23.8)
CRP, (n = 157), mg/dL	119 (218.7)
***Previous bDMARD therapies before TCZ initiation, n (%)***	
0	52 (28.4)
1	64 (35.0)
2	40 (21.9)
3+	27 (14.8)

SD: standard deviation; BMI: Body mass index; RA: rheumatoid arthritis; TCZ: tocilizumab; GI: gastrointestinal; TJC: tender joint count; SJC: Swollen joint count; RF: rheumatoid factor; ACPA: anti-citrullinated protein antibodies; DAS-28: disease activity score for 28 joint count; ESR: erythrocyte sedimentation rate; CRP: C-reactive protein; bDMARDS: biologic disease modifying antirheumatic drugs.

The major comorbidities recorded at baseline were hyperlipidaemia (54.1%), and cardiovascular-related disorders (37.2%). On average patients had 10.9 tender joint counts (TJC) and 8.0 swollen joint counts (SJC) and a mean 5.8 DAS28-ESR score. About one third (28.4%) of the patients had no prior bDMARD exposure and 71.6% received at least one biologic therapy before TCZ initiation.

### Persistence to/Discontinuation from TCZ therapy

**Figure 1.**
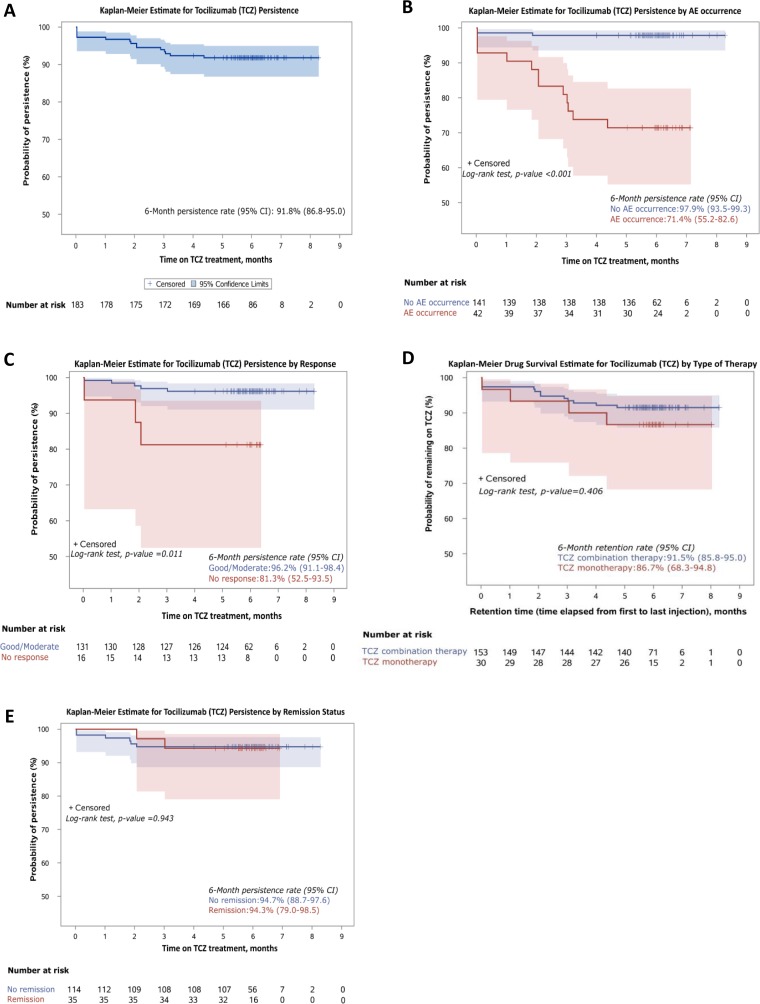
Kaplan-Meier estimate of persistence to Tocilizumab therapy at 6 months of A) the whole cohort and by B) adverse event occurrence, C) response, D) type of therapy, E) remission status.

Out of the 183 patients who received TCZ therapy, 85.8% received 6 infusions while 72.1% received 7 infusions within the first 6 months. K-M survival probability estimates for persistence to TCZ therapy was 91.8% (*Figure 1*). Parameters that affected persistence were the occurrence of adverse events (AEs) (p<0.0001) and treatment response (p=0.0219), whereas type of TCZ therapy (p = 0.9388) and remission status (p = 0.9435) were not (*Figure 1*).

We sought to investigate whether any patient (age, gender, BMI, RF/anti-CCP status) or treatment (type of TCZ therapy, number of prior bDMARD therapy) attributes were prognostic factors for TCZ therapy discontinuation. Univariate cox regression analysis did not result in any significant association of the tested characteristics to TCZ therapy discontinuation (*Table 2*). As expected, multivariate analysis of the above variables did not produce any significant result on therapy discontinuation. Similarly, neither univariate nor multivariate cox regression analysis revealed a significant association between the same characteristics and persistence to TCZ therapy (data not shown).

**Table 2.** Univariable Cox regression models for the association of selected factors with TCZ discontinuation rate.

**Parameter**	**Category vs. reference**	**n_pt_**	**n_events_**	**HR**	**(95%CI)**	**p-value**
**Age (years)**	18–60 vs >60	104 vs 79	7 vs 10	0.52	(0.20–1.35)	0.179
**Gender**	males vs females	22 vs 161	0 vs 17	0.20	(0.01–3.59)	0.275^a,b^
**BMI (kg/m^2^)**	<25 vs ≥25	56 vs 127	6 vs 11	1.25	(0.46–3.39)	0.657
**RF/anti-CCP status**	RF(−) and anti-CCP(−) vs RF(+) and/or anti-CCP(+)	34 vs 130	3 vs 13	0.89	(0.25–3.14)	0.861
**Type of TCZ therapy**	TCZ monotherapy vs TCZ combination therapy	30 vs 153	4 vs 13	1.60	(0.52–4.90)	0.412
**Prior bDMARD therapy**	0–1 vs ≥2	116 vs 67	8 vs 9	0.50	(0.19–1.29)	0.152

a Firth’s correction was used due to zero events in male patients.

b Proportional hazards assumption was violated.

CI: confidence interval; HR: hazard ratio.

### Efficacy and safety

**Figure 2.**
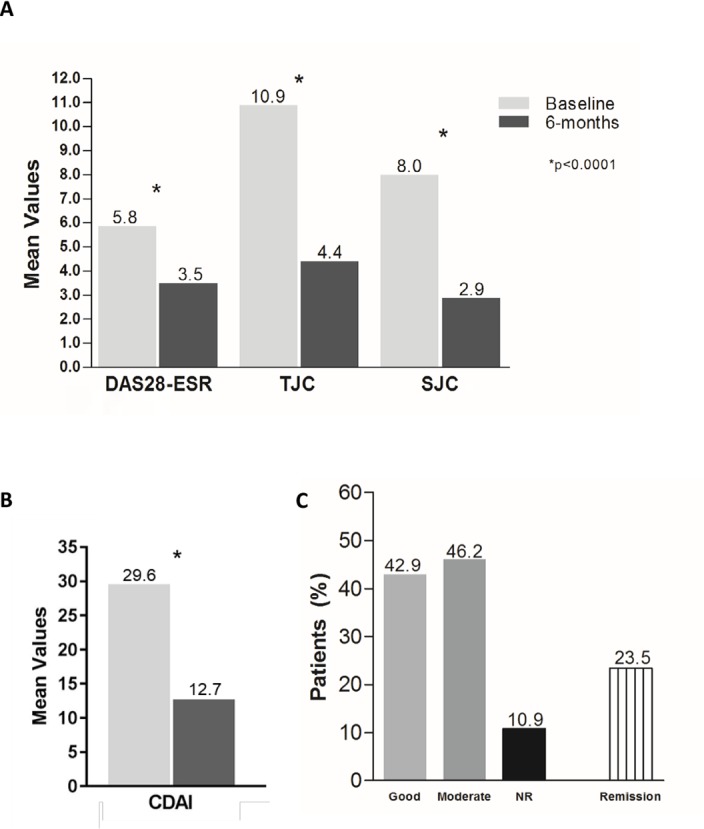
Efficacy of Tocilizumab therapy at 6 months. Mean A) DAS28-ESR, Tender Joint Counts (TJC) and Swollen Joint Counts (SJC) and B) CDAI, values at baseline and 6 months after Tocilizumab therapy. C) Percentage of patients achieved Good and Moderate response and Remission rates. NR: No response.

The efficacy of TCZ therapy was measured in terms of reduction in the DAS28-ESR, CDAI, TJC and SJC from baseline to 6 months, as well as the percentage of patients that achieved EULAR Good/moderate response rates. Six months after initiation of TCZ therapy there was −1.3 reduction in the DAS28-ESR score, from 5.8 (95%CI, 5.7 – 6.0) to 3.5 (95%CI, 3.3 – 3.7), the mean number of TJC dropped from 10.9 (95%CI, 10.0 – 11.8) at baseline to 4.4 (95%CI, 3.7 – 5.1) at 6 months and respectively, the mean number of SJC from 8.0 (95%CI, 7.1 – 8.9) to 2.8 (95%CI, 2.3 – 3.4) (*Figure 2A*). CDAI score dropped from 29.6 (95%CI, 27.6 – 31.5) at baseline to 12.7 (95%CI, 11.1 – 14.3) at 6 months (*Figure 2B*). The clinical benefit in all parameters assessed was statistically significant. In terms of EULAR responses, 89.1% of the patients achieved Good/Moderate response and 23.5% achieved remission (DAS28<2.6) (*Figure 2C*).

Forty-two patients (23.0%) experienced at least one AE, with 31 experiencing 1, 7 experiencing 2, 3 experiencing 3 and 1 experiencing 4, during follow-up period. In total, 58 AEs were recorded, 38 (65.5%) of which were mild, 12 (20.7%) were moderate, 2 (3.4%) were severe and 6 (10.3%) were of unknown severity. In total, 58 AEs were recorded, with 40 being related to TCZ out of which none was severe. The majority of the AEs were mild (24) or of moderate severity (10), while 6 were of unknown severity. while 6 were of unknown severity. Of note, the 2 severe events that were recorded in the study (adenocarcinoma of the uterus and a hip fracture) were not related to TCZ therapy according to the judgment of the treating rheumatologist. Most common AEs were infections, hematologic and musculoskeletal disorders and hyperlipidaemia (*Table 3*). Three hypersensitivity reactions were attributed to TCZ therapy, but they were all mild in nature and none led to permanent drug discontinuation. Interestingly, although 4.4% of the patients had a history of gastrointestinal (GI) perforation/ulceration/diverticulitis, no signs of perforation were recorded during the observational period. Only 11 patients (6.0%) discontinued treatment due to a related AE (3 due to lack of efficacy, 3 due to haematological disorders, 3 due to musculoskeletal disorders, 1 due to oral thrush and 1 due to a skin disorder).

**Table 3.** Summary of Adverse Events.

**Event**	**Related to TCZ therapy**	Not related to TCZ therapy^*^	**Total**
**Infections (total)**	9	3	12
*Urinary tract infection*	3	2	5
*Upper respiratory tract infection*	3	1	4
*Skin infection*	3	0	3
**Hematologic disorders (total)**	10	0	10
*Leucopoenia*	3	0	3
*Neutropoenia*	5	0	5
*Thrombocytopoenia*	2	0	2
**Musculoskeletal disorders**	4	4	8
**Metabolic disorders**	5	0	5
*Hyperlipidaemia*	5	0	5
**Hypersensitivity reactions**	3	1	4
**Lack of efficacy**	3	0	3
**Skin disorders**	2	2	4
**Gastrointestinal disorders**	2	2	4
**Cardiovascular Events (total)**	0	1	0
*Transient ischemic attack*	0	1	0
**Nervous system disorders**	0	2	2
**Tumours**	0	1	1
*Adenocarcinoma of uterus*	0	1	1
**Other**	2	2	4

**Total**	**40**	**18**	**58**

* Not related to TCZ therapy according to the judgment of the treating rheumatologist.

### Adherence to therapy

Adherence to therapy was monitored on the time each infusion was conducted based on the treatment schedule for each individual patient according to the local SPC. Seventy-seven patients (42.1%) had no delay in their scheduled TCZ infusion compared to 106 patients (57.9%) who had at least 1 dose delay. The majority of the non-adherent patients (75%) had 1 or 2 dose delays, whereas <5% of the patients had 4 or more infusion delays. The overall mean adherence for every scheduled TCZ infusion was 79% (range 73% – 83%). The reasons for non-adherence to the scheduled infusions are shown in *Table 4*. Only 15.9% of these reasons were medically related (concurrent interventions for other medical conditions and AE). The other 84.1% of the reasons were non-medically related, with the most predominant being issues with drug supply (40%), personal reasons (16%) and interference with social habits, mainly holidays (16%).

**Table 4.** Reasons of non-adherence to at least 1 TCZ infusion.

**Reason**	**Patients, n (%)**
**Medically related**	
*Other medical interventions^‡^*	*5 (5.7)*
*Adverse Event*	*9 (10.2)*
**Total**	**14 (15.9)**

**Non medically related**	
*Drug supply issues*	*35 (39.8)*
*Personal reasons*	*14 (15.9)*
*Holidays/administrative reasons*	*14 (15.9)*
*Patient absence/negligence*	*5 (5.7)*
*Unknown*	*6 (6.8)*
**Total**	**74 (84.1)**

‡ Scheduled surgery (4), Endometrial biopsy (1)

## DISCUSSION

The aims of this study were to investigate persistence and adherence to TCZ therapy in routine clinical practice in the setting of the private rheumatology practitioners. We found the 6-month persistence rate to be at 92%, which is in line with other RWD phase IIIb studies.^16–18^ Nevertheless, even these studies sometimes do not represent daily clinical practice. Indicative sources of daily clinical practice such as the Danish and Swedish registry show persistence to be around 80% and 79% respectively, whereas in our study persistence was much higher.^19,20^ Persistence to TCZ therapy seems to be affected by a variety of factors. In our case, persistence to therapy was affected by the occurrence of an adverse event and the achievement of a response, in line with the findings of a European collaborative study.^21^ Several studies showed that low levels of initial CRP, bDMARD history and TCZ monotherapy treatment are potential predictors of TCZ discontinuation.^20–22^ In contrast, none of the factors analysed in our study was able to show any predictive potential for TCZ discontinuation. This could be attributed to the short follow up period as well as the small number of patients. Furthermore, the ratio of patients receiving TCZ monotherapy vs TCZ combination is roughly 1:10, thus adding more difficulty in the statistical modelling. TCZ monotherapy was approved locally only a few months before the study commenced, therefore it was not expected that many patients will initiate TCZ monotherapy during study enrolment.

In terms of efficacy, an indirect comparison with the similar type RWD studies ROUTINE,^17^ TAMARA^16^ and ACT-SURE^18^ reflects mixed results. About 90% of the patients in REMISSION II had a Good/moderate EULAR response, similar with the responses in ROUTINE and a higher than TAMARA (75%) at week 24. In contrast, in REMISSION II, a −1.3 DAS-ESR decrease was observed from baseline within 6 months, whereas in ROUTINE and TAMARA, DAS28 changes where −2.7 and −3.4 respectively in the first 24 weeks. Similarly, to DAS28 decrease, the percentage of patients who achieved remission was 23.5% in REMISSION II which is lower, than the percentages in ROUTINE (45.7%), TAMARA (47.6%) and ACT-SURE (56.8%) at 24 weeks. This comparison should be interpreted with caution, as the percentages of patients who were TNF-naïve in those studies are twice as much that the ones in REMISSION II. Such difference in the patient populations could have accounted for the low decrease in DAS28 and remission rates observed in our study.

The main reasons for non-adherence to TCZ therapy were non-medically related with the most frequent being lack of TCZ availability in domestic pharmacies. In general, the private practice setting can differ from large hospital clinics since there is harder control of the scheduled infusion intervals. Several reasons could attribute for a patient to miss or delay his/her scheduled infusion. These reasons could vary depending on a country’s healthcare system and the level of access. For instance, in the US, retail pharmacies could be associated with higher non-adherence.^22^ In a recent study conducted in Greece, ninety-two percent of rheumatologists and 96% of pharmacists confirmed that patients experience difficulties in accessing RA medication and the most frequently reported barriers to access pharmaceutical treatment were difficulties in the prescription process, distance from central supply pharmacies and medicine shortages in hospitals.^23^ Such problems were frequent when austerity measures rapidly decreased public healthcare spending since the beginning of the Greek economic crisis.

The safety profile of TCZ was comparable to what has been previously observed.^24^ The most common, as expected, AEs were infections. A total of 58 AEs (31.7%) were recorded with forty of them being related to TCZ. Only 2 severe AEs (SAEs) were recorded with none being related to TCZ. It is of note that the rate of SAEs is very low in this study in contrast with the high rate treatment related AEs.^18^ The nature of the study and the limitations of its setting (retrospective, private practice) could account for these differences. Interestingly, we did not observe any incidence of GI perforation, although 63% of the patients were receiving concomitant corticosteroids while being treated with TCZ. This finding is likely attributed to the adequate administration of proton-pump inhibitors.

These are some limitations in this study which need to be considered. This study was retrospective mainly measuring the persistence to TCZ therapy for a 6-month time interval. Thus, the effect of therapy persistence was not evaluated in the long term, neither its implication in terms of adherence and discontinuation rates. However, in regards to its primary endpoint, this study demonstrated a very good persistence rate of TCZ therapy. Medical reasons of non-persistence to TCZ therapy were relatively low. It is evident that the absence of non-medical reasons such as drug supply issues could have led to even higher therapy persistence rates thus improving performance of TCZ treatment in a real-world setting. This should also be taken into account by the National Healthcare System when assessing health outcomes of any available therapeutic approach.

In conclusion, persistence to TCZ appeared to be high in the rheumatology private practice setting in Greece and no adherence to medication is attributed mainly to non-medical reasons.

## REMISSION II investigators

A. Aggelou, A. Akkizidou, P. Alexiou, A. Andrianakos, A. Antoniou, D. Deliotzaki, A. A. Drosos, A. Kandili, E. Kaskani, P. Katzakis, E.M. Koukli, G. Koukouvitakis, A. Lagoudakis, M. Mamalaki, T.E. Markatseli, D. Mavridou, M. Papadopoulou, M. Patriki, D. Petrou, A. Siagkri, D. Soukera, A. Theodoridou, E. Triantafillidou, G. Xirogiannis, M. Zakalka.
